# Garlic augments the functional and nutritional behavior of *Doenjang*, a traditional Korean fermented soybean paste

**DOI:** 10.1038/s41598-019-41691-3

**Published:** 2019-04-01

**Authors:** Ashutosh Bahuguna, Shruti Shukla, Jong Suk Lee, Vivek K. Bajpai, So-Young Kim, Yun Suk Huh, Young-Kyu Han, Myunghee Kim

**Affiliations:** 1 Department of Food Science and Technology, Yeungnam University, Gyeongsan, Gyeongsangbuk-do 38541 Republic of Korea; 2 Department of Energy and Materials Engineering, Dongguk University-Seoul, 30 Pildong-ro 1-gil, Seoul, 04620 Republic of Korea; 3 Division of Food & Nutrition and Cook, Taegu Science University, Daegu, 41453 Republic of Korea; 4 Department of Agrofood Resources, National Institute of Agricultural Sciences, Rural Development Administration, 166 Nongsaengmyeong-ro, Iseo-myeon, Wanju, Jellabuk-do 55365 Republic of Korea; 5 Department of Biological Engineering, Biohybrid Systems Research Center (BSRC), Inha University, 100 Inha-ro, Nam-gu, Incheon 22212 Republic of Korea

## Abstract

Three different forms of garlic, namely, fresh garlic (2%, 6%, 10%), heat-dried (1%, 2%, 3%) and freeze-dried (1%, 2%, 3%), were supplemented in soybean paste to prepare *Doenjang* and further evaluated for functional, nutritional and safety aspects. Results showed a considerable antioxidant and anti-proliferative activity of garlic-supplemented *Doenjang*. As a measure of nutritive value, a high amount of total free amino acids, 4,290.73 mg/100 g–5,492.94 mg/100 g, was observed in prepared *Doenjang*. Among all preparations, 3% freeze-dried garlic-supplemented *Doenjang* proved the most effective against gastric adenocarcinoma and lung adenocarcinoma with 50% inhibition concentration of 7.66 ± 0.53 mg/mL and 7.82 ± 0.34 mg/mL, respectively. However 10% fresh-garlicsupplemented *Doenjang* (GGD-10) showed better activity against colorectal adenocarcinoma (HT29) cell line. Furthermore, GGD-10 effectively reduced colony formation and altered mitochondrial membrane potential of HT29 cells. Absence of pathogenic bacteria (*Staphylococcus aureus, Salmonella* species and *Bacillus cereus*) and aflatoxin was observed in *Doenjang* samples. In addition, nontoxic amount of anti-nutritional biogenic amines was observed in all the samples. The results collectively suggest that the addition of garlic in *Doenjang* can improve its nutritional and functional value, resulting in the protection of consumers from protein deficiencies and various stress conditions.

## Introduction

Soybean (*Glycine max*) is a major crop long being used as a chief source of protein in Asian countries. Besides proteins, it is a rich source of minerals and vital phytochemicals such as isoflavonoids^1^. Owing to its high nutritious value, its consumption has increased many folds worldwide in the recent time. Soybeans are the most important legume that contains all the essential amino acids required for human development and can be consumed as such (seeds) or used after processing in the form of soy flour, defatted soy flakes, soy proteins concentrate and soy protein isolates^2^. Apart from this, it is also consumed in the form of fermented foods such as *Kochujang*, *Chungukjang*, *Doenjang*, soy sauce^1^, *Sufu*^3^, *Aakhone* and *Peruyaan*^4^. In particular, *Doenjang* is the most common fermented soy product in Korea with numerous beneficial activities including antioxidant, anti-inflammatory, anticancer and antimutagenic properties^5^.

Traditionally, *Doenjang* is prepared using fermented *Meju* and brine (salt solution). Typically, *Meju* is prepared by soaking, cooking, and crushing soybeans, followed by modelling into solid blocks of defined shape and size where consortia of naturally occurring microbes (bacteria and moulds) grow with the time. Subsequently, salt solution is added to *Meju* and fermented for few more months. Later, the solid portion is separated from the liquid and fermented for several months resulting in *Doenjang*^6,7^. The nutrient value, taste and texture of *Doenjang* is highly dependent on fermentation conditions, basic ingredients and involvement of microorganisms^1,5^. Hence, the addition of new ingredients has high possibilities to improve the *Doenjang* quality both in taste and functional property. Further, selection of superior quality of basic ingredients (for example, seeds) also have an impact on *Doenjang* quality which can be achieved by utilizing many modern techniques^8^. Extensive efforts have been made to improve the *Doenjang* quality by altering fermentation condition, substrate and microbial inoculum^9,10^.

In our previous study, we successfully made the different microbes-based starter culture, along with the variation in the fermented material composition, to achieve the *Doenjang* with novel properties that proved efficient to limit the aflatoxin, biogenic amines and pathogenic microbes well below the acceptable level^1,6,11^. *Doenjang* is vulnerable to fouling by aflatoxins, biogenic amines and *Bacillus cereus* which adversely affects human health^6,11^. Particularly, biogenic amines are the most common contamination in soybean paste during fermentation, excessively high levels of which are associated with serious health issues^6^. More importantly, these biogenic amines are converted into nitrosamine and behave like a carcinogen^12^. Concerning to this, the present study was designed to prepare *Doenjang* by adding various forms (fresh, heat-dried and freeze-dried) and combinations (1%, 2%, 3%, 6% and 10%) of garlic with soybean paste and evaluated for its physicochemical (color, pH, moisture, salt and ash), nutritional and functional properties, along with determination of toxicological properties by quantifying biogenic amines, aflatoxins and presence of food pathogenic bacteria.

## Results and Discussion

### Physiochemical analysis of the prepared *Doenjang*

Garlic is a storehouse of many important phytochemicals, mainly organosulfur compounds (allicin, alliin, *S*-allylcysteine or *S*-allyl mercaptocysteine), and has multiple health benefits^13^. Several clinical studies suggested its apparent role in hepatoprotection, immunomodulation, atherosclerosis, lipid metabolism, blood pressure maintenance and regulation of diabetes and cardiovascular diseases^13,14^. In the present study, nine different types of *Doenjang* were prepared using three forms of garlic (Table 1, Supplementary Fig. S1) and examined for its physicochemical analysis. Initially, pH was examined, which is considered as the most important factor of the fermented foods and vital to commence many biochemical activities. It not only provides a lucrative environment for many biochemical reactions but also acts as a barrier for many microbial contamination^6^. Results revealed the slight acidic pH in all *Doenjang* samples between 5.17 ± 0.04 and 6.12 ± 0.02 (Table 2). Highest acidic pH was observed in FGD-2 (5.17 ± 0.04), while the least was in the GGD-2 (6.12 ± 0.02). All the garlic-supplemented samples, except GGD-2 and HGD-1, have a slightly lower pH as compared to control. It is well established fact that acidic pH is an indication of microbial fermentation; hence, results insight that the decreased pH in garlic-supplemented samples is possibly because of good fermentation of soybean paste. The moderately acidic pH in the *Doenjang* samples attests it safe for consume, contrary to the excessively low pH which adversely affects health by causing acidosis. The results are consistent with previous findings, signifying the role of starter culture and alterations in processing conditions and ingredients on the pH of prepared *Doenjang*^6,15^.

**Table 1:** List of *Doenjang* samples prepared with various combinations of garlic.

Proportion and form of garlic added to soybean paste	Abbreviation of garlic added *Doenjang* sample
2% ground garlic	GGD-2
6% ground garlic	GGD-6
10% ground garlic	GGD-10
1% heat-dried garlic	HGD-1
2% heat-dried garlic	HGD-2
3% heat-dried garlic	HGD-3
1% freeze-dried garlic	FGD-1
2% freeze-dried garlic	FGD-2
3% freeze-dried garlic	FGD-3
No garlic-added soybean paste	Control (no garlic added *Doenjang*)

**Table 2:** Physiochemical analysis and surface color value of *Doenjang* samples prepared with various form of garlic.

*Doenjang* sample	pH	Salinity (%)	Moisture (%)	Ash (%)	Hunter’s value		
					L*	a*	b*
Control	5.83 ± 0.07^b^	13.21 ± 0.52	62.07 ± 5.36	14.12 ± 0.13^a,b^	38.28 ± 0.65^b^	6.39 ± 0.46^b,c^	11.84 ± 0.83^c,d^
GGD-2	6.12 ± 0.02^a^	14.36 ± 1.37	59.28 ± 3.76	13.91 ± 0.16^b,c^	38.46 ± 0.29^b^	9.93 ± 0.81^a^	14.72 ± 1.44^a^
GGD-6	5.72 ± 0.02^c^	13.98 ± 1.38	60.02 ± 4.44	13.58 ± 0.16^d^	38.94 ± 0.76^a,b^	5.32 ± 0.57^c^	12.55 ± 0.04^b,c^
GGD-10	5.40 ± 0.06^e^	13.86 ± 0.13	60.45 ± 5.39	13.27 ± 0.17^e^	38.08 ± 0.95^b,c^	5.38 ± 1.35^c^	13.67 ± 0.68^a,b^
HGD-1	5.85 ± 0.05^b^	13.12 ± 0.32	55.09 ± 3.22	14.21 ± 0.19^a^	36.43 ± 0.57^d^	5.81 ± 0.99^b,c^	8.67 ± 0.94^f^
HGD-2	5.73 ± 0.02^c^	12.97 ± 0.98	59.56 ± 4.88	13.51 ± 0.20^d,e^	35.30 ± 0.89^e^	6.59 ± 0.98^b,c^	8.90 ± 0.98^f^
HGD-3	5.80 ± 0.05^b^	14.15 ± 0.01	59.05 ± 3.97	13.54 ± 0.07^d^	36.63 ± 1.02^d^	5.65 ± 0.67^b,c^	6.79 ± 0.67^g^
FGD-1	5.74 ± 0.03^c^	13.87 ± 0.88	57.98 ± 5.82	13.58 ± 0.15^d^	37.16 ± 0.60^c,d^	5.73 ± 0.31^b,c^	10.16 ± 2.35^e,f^
FGD-2	5.17 ± 0.04^f^	13.32 ± 0.71	59.74 ± 4.95	13.59 ± 0.13^d^	39.54 ± 0.49^a^	6.80 ± 0.09^b^	10.83 ± 0.30^d,e^
FGD-3	5.51 ± 0.02^d^	13.54 ± 0.60	57.60 ± 3.88	13.71 ± 0.22^c,d^	36.60 ± 0.39^d^	6.07 ± 0.71^b,c^	10.67 ± 0.38^d,e^

Each value represents mean ± SD of three independent experiments. L* represents lightness in the scale 0 to 100 (black: 0, white: 100), a* represents redness (green: −60, red: 60) and b* represents yellowness (blue: −60, yellow: 60). Mean values with different lower case letters (a–f) in a column are significantly different (*P* < 0.05).

In addition to pH, salinity, ash and moisture content are important parameters to determine the fermented food quality. Sodium chloride is the most common food additive that not only acts as a food preservative but also improves taste, texture and shelf-life^16^. We observed salinity in the range of 12.97 ± 0.98%–14.36 ± 1.37% (Table 2). Minimum salinity was observed in HGD-2 (12.97 ± 0.98%), whereas maximum in GGD-2 (14.36 ± 1.37%). A negative correlation between salinity and increasing amount of fresh garlic (*y* = −0.06*x* + 14.44, *r*^2^ = 0.91) was observed in the prepared *Doenjang*.

We further evaluated the moisture content, which is directly associated with texture, appearance, weight and shelf-life of foodstuff, along with a supportive role in microbial growth that accelerates the fermentation. Highest moisture content was observed in the control (62.07 ± 5.36%), whereas the lowest in HGD-1 (55.09 ± 3.22%) (Table 2). Also, a positive correlation was observed in the moisture content and amount of fresh ground garlic added *Doenjang* (*y* = 0.15*x* + 59.03, *r*^2^ = 0.97).

Next, we determined the ash content, which reflects the mineral content of the sample. The ash mainly consists of K, Na, Mg and Ca, along with trace amounts of Al, Fe, Cu, Mn and Zn. All these minerals have a significant role in human development and also act as cofactor of many proteins^17^ and thus are very important to supply from the foods. We observed the ash content in the range of 13.27 ± 0.17%–14.21 ± 0.19% (Table 2). Maximum ash content was detected in HGD-1 (14.21 ± 0.19%), whereas the minimum content was in GGD-10 (13.27 ± 0.17%).

Food color is an important factor that determines its appearance and overall consumer acceptability. Surface color of all samples was determined using a colorimeter; minimum lightness (L*) value was reported for HGD-2 (35.30 ± 0.89) and the maximum for FGD-2 (39.54 ± 0.49), whereas the control group showed L* of 38.28 ± 0.65 (Table 2). In contrast, maximum and minimum redness (a*) values were noticed in GGD-2 (9.93 ± 0.81) and GGD-6 (5.32 ± 0.57) samples, respectively, and maximum and minimum yellowness (b*) values were noticed in GGD-2 (14.72 ± 1.44) and HGD-3 (6.79 ± 0.67), respectively. It is difficult to generalize the color change, however, variation of the color gives idea of the involvement of the diverse microbes that act on the complex biomolecules (starch and proteins) and degrade them into simple amino acids and sugars responsible for unique aroma, color and taste^6^. Results corroborated those of a recently published report on color change in *Doenjang* prepared using various starter cultures with varied ingredients^6^.

### Determination of free amino acids

Total free amino acids in all prepared *Doenjang* were quantified, and the results are depicted in Table 3. An important role of amino acids in human metabolism as a building block of proteins, a stabilizer of nucleic acid and growth factor is well mentioned in the literature^18^. Besides the nutritive value, free amino acids are also responsible for color and taste of the food^6^. Owing to many benefits of amino acids, we quantified and characterized free amino acids in the prepared *Doenjang* and observed a high value between 4,290.73 mg/100 g and 5,492.94 mg/100 g (Table 3) (Supplementary Figs S2–S11). This high total free amino acid content is indicative of good fermentation and involvement of diverse microbial proteases, which is well supported by the results of previous reports, suggesting the importance of proteolysis during fermentation of soy products^6^.

**Table 3:** Quantitative analysis of total free amino acids in *Doenjang* samples prepared with various forms of garlic (mg/100 g).

	Amino acid	*Doenjang* samples									
		Control	GGD-2	GGD-6	GGD-10	HGD-1	HGD-2	HGD-3	FGD-1	FGD-2	FGD-3
Essential amino acids	Isoleucine	295.22	304.14	310.53	357.70	298.89	342.39	350.92	313.59	295.32	365.34
	Leucine	427.41	450.29	456.25	556.96	442.42	500.03	530.34	465.5	437.95	559.26
	Lysine	328.16	324.13	349.55	373.22	335.55	407.78	356.69	353.73	327.64	403.74
	Methionine	71.24	65.03	69.78	83.96	68.11	77.92	77.41	67.10	65.47	81.20
	Phenylalanine	266.46	284.67	310.20	308.62	294.15	312.98	315.03	313.10	281.29	349.33
	Valine	294.81	292.14	303.88	330.01	283.00	329.17	327.78	314.42	297.18	366.32
	Total	1,683.30	1,720.40	1,800.19	2,010.47	1,722.12	1,970.27	1,958.17	1,827.85	1,704.85	2,125.19
Non-essential amino acids	Arginine	15.97	45.63	137.01	29.58	49.44	49.28	36.49	86.33	45.07	150.71
	Proline	185.24	178.04	201.73	185.80	168.09	177.18	196.68	202.46	172.78	216.34
	Tyrosine	65.29	125.13	191.88	72.58	140.42	180.53	64.34	161.89	60.05	148.40
	Glycine	146.35	143.04	148.92	148.22	133.48	169.90	152.31	144.60	134.42	160.48
	Alanine	509.38	349.16	385.19	492.57	388.16	360.68	513.14	372.14	429.20	362.87
	Serine	248.13	226.15	269.69	265.87	253.60	265.68	289.11	291.89	239.93	316.13
	Glutamic acid	759.97	706.36	802.67	817.49	738.00	934.64	860.75	819.53	739.45	880.77
	Aspartic acid	99.61	344.98	325.59	181.07	232.73	427.10	167.59	336.71	187.81	458.66
	Total	2,029.94	2,118.49	2,462.68	2,193.18	2,103.92	2,564.99	2,280.41	2,415.55	2,008.71	2,694.36
Non-proteinogenic amino acids	Ornithine	196.29	176.12	124.67	172.95	163.86	147.00	216.06	163.30	195.18	143.00
	*O*-Phosphoserine	21.75	24.30	26.18	27.61	24.43	32.82	33.15	25.06	22.04	31.95
	Taurine	11.88	11.79	13.14	12.99	11.61	18.05	15.57	16.25	11.35	18.45
	*β*-Aminoisobutyric acid	187.83	184.84	217.50	199.29	202.54	231.90	222.46	229.12	185.98	248.37
	Sarcosine	14.47	18.87	18.08	23.64	15.94	18.24	18.84	17.01	25.76	22.83
	*α*-Amino adipic acid	48.90	50.96	48.40	63.95	53.86	71.46	61.12	53.00	56.08	63.60
	L-Citrulline	31.80	42.03	54.76	36.54	57.91	84.01	34.63	61.89	42.72	78.93
	*γ*-Aminobutyric acid	28.24	27.55	30.85	33.94	26.83	26.70	32.87	29.26	29.60	34.39
	Ethanolamine	21.75	18.50	19.05	22.81	20.02	24.01	21.84	21.69	20.95	24.21
	Hydroxylysine	14.58	7.99	7.78	10.46	10.90	10.56	11.94	5.32	5.92	7.66
	**Total**	**577.49**	**562.95**	**560.41**	**604.18**	**587.9**	**664.75**	**668.48**	**620.93**	**595.58**	**673.39**
**Grand total**		**4,290.73**	**4,401.84**	**4,823.28**	**4,807.83**	**4,413.94**	**5,200.01**	**4,907.06**	**4,863.91**	**4,309.14**	**5,492.94**

We further characterized free amino acids as proteinogenic and non-proteinogenic and observed the highest proteinogenic amino acid content in FGD-3 (4,819.55 mg/100 g), followed by HGD-2 (4,535.26 mg/100 g) and the least in the control (3,713.24 mg/100 g). Furthermore, proteinogenic amino acids were regrouped into essential and non-essential amino acids. The outcome of the study suggests the higher essential amino acids in FGD-3 (2,125.19 mg/100 g), signifying its good health effect (Table 3). When compared with the control, FGD-3 showed 1.26-fold enhanced essential amino acid content. Among the all estimated essential amino acids, leucine was predominated with the highest value in FGD-3 (559.26 mg/100 g), followed by GGD-10 (556.96 mg/100 g), signifying 30.8% and 30.3% augmentation, respectively, as compared to control. Moreover, the overall high level of non-proteinogenic amino acids was observed in FGD-3 (673.39 mg/100 g), representing 1.16-fold higher values as compared to control (577.49 mg/100 g) (Table 3).

Accumulating results suggest the indispensable biological role of non-proteinogenic amino acids, such as involvement of taurine with membrane stabilization, osmoregulation, modulation of Ca^+2^ signaling and essential of cardiovascular function, development and function of the central nervous system^19^. We detected taurine highest amount in FGD-3 (18.45 mg/100 g) and least in the FGD-2 (11.35 mg/100 g). In addition, we observed high level of *β*-aminoisobutyric acid which induces the expression of thermogenic genes, resulting in browning of white fat cells^20^, and is effective against obesity^21^. The maximum level of *β*-aminoisobutyric acid was detected in FGD-3 (248.37 mg/100 g), which was 1.3-fold higher than that in the control (187.83 mg/100 g) (Table 3). Results collectively showed good amount of essential and non-proteinogenic amino acids in all the garlic-supplemented *Doenjang* and more precisely in FGD-3 and thus could be effective to overcome protein deficiency if consumed regularly.

We speculated that the deviation in the amount of total free amino acid in different samples is directly related with the variation of the raw material (different form and proportion of garlic) that leads fermentation through a diverse environment with different proteolytic events. The findings are supported by the previous report^6^ representing the effect of various starter culture which leads to modulating the fermentation and diversification of free amino acids in *Doenjang*. However, unlike to this research, a lesser amount of proteinogenic amino acids was noticed in the various *Doenjang* samples^6^. Apart from nutritive value, amino acids are also responsible for the flavor to particular food material, therefore, we also categorized free amino acids based on their taste. Broadly amino acids can be classified into four major taste groups such as sweet taste (lysine, glycine, alanine, serine, threonine), umami taste (glutamic acid, aspartic acid, cysteine), bitter taste (isoleucine, leucine, methionine) and other taste (phenylalanine, valine, arginine, proline, tyrosine, histidine)^6^. We observed a high amount of total sweet taste amino acids in HGD-3 (1,311.25 mg/100 g), while least in GGD-2 (1,042.48 mg/100 g) (Supplementary Fig. S12). The highest amount for umami taste amino acids were detected in FGD-3 (1,339.43 mg/100 g), while least in the control (859.58 mg/100 g) (Supplementary Fig. S12). Similarly, the maximum and minimum amount of bitter taste and other taste amino acids were detected in FGD-3 and control, respectively (Supplementary Fig. S12).

### Estimation of phenolics, flavonoids and antioxidant potential of *Doenjang*

Diseases such as cancer, cataract, rheumatoid arthritis and neurodegenerative ailments such as Alzheimer’s disease and Parkinson’s disease are associated directly with the oxidative stress elevated by various reactive oxygen species (ROS)^22^. The cellular antioxidant machinery scavenges the ROS, although sometimes it is insufficient for scavenging it to the basal level particularly when large amounts of ROS are generated^22^. In such condition, exogenous antioxidants are required to scavenge the excessive ROS. As external antioxidant, herbs provides more promise due to its good efficiency and safety in nature unlike to synthetic compound. There are number of studies reporting the health beneficial potential of plants due to its high antioxidant activities. Phenolics and flavonoids are the important constituents of plants responsible for numerous biological activities including antioxidant activity. Moreover, a positive correlation of phenolics and flavonoids with antioxidant activities has been well documented^23^. A comparative total phenolics and flavonoid content in the *Doenjang* are depicted in Table 4, which revealed the higher phenolics content in the garlic-supplemented samples than in the control. The highest phenolics content was observed in the FGD-3 (35.14 ± 2.29 mg gallic acid equivalents (GAE)/g). Unlike phenolics, higher flavonoid content was observed in GGD-2 (4.25 ± 1.13 mg quercetin equivalents (QE)/g), followed by GGD-6 (4.17 ± 1.13 mg QE/g) and FGD-3 (4.14 ± 1.10 mg QE/g). Results are assisted with the number of reports presenting the diverse amount of phenolics and flavonoids in the soy fermented food^24,25^. In a recent study, variations in phenolic and flavonoid contents were reported in *Doenjang* formed by the addition of different plant materials^15^.

**Table 4:** Quantification of total phenolics, flavonoids and antioxidant potential of *Doenjang* prepared with various combinations of garlic.

*Doenjang* sample	Total phenolic content (mg GAE/g)	Total flavonoid content (mg QE/g)	IC_50_ (mg/mL)			Ferric reducing antioxidant power assay (mmol Fe^+2^/g)
			DPPH^●^ scavenging	ABTS^●+^ scavenging	Superoxide anion scavenging	
Control	27.50 ± 3.72	4.07 ± 1.12	42.90 ± 4.25^a^	6.30 ± 0.51^a^	23.32 ± 3.06^c,d^	2.91 ± 0.05^c^
GGD-2	31.60 ± 1.82	4.25 ± 1.13	22.93 ± 0.62^c,d^	5.99 ± 0.09^a,b^	28.41 ± 2.98^b,c^	3.42 ± 0.15^a,b,c^
GGD-6	28.82 ± 1.73	4.17 ± 1.13	30.42 ± 1.18^b^	5.54 ± 0.17^a,b,c^	29.15 ± 4.52^b,c^	3.75 ± 0.23^a,b^
GGD-10	30.92 ± 1.44	4.10 ± 1.20	28.83 ± 0.72^b,c^	5.59 ± 0.29^a,b,c^	20.82 ± 0.83^d^	3.60 ± 0.02^a,b,c^
HGD-1	30.22 ± 1.01	3.55 ± 1.04	40.34 ± 6.27^a^	5.39 ± 0.08^b,c^	23.98 ± 1.59^c,d^	3.24 ± 0.07^b,c^
HGD-2	34.82 ± 2.33	3.71 ± 0.93	20.39 ± 0.55^d^	5.94 ± 0.07^a,b^	30.97 ± 2.76^b^	3.98 ± 0.20^a^
HGD-3	34.43 ± 4.12	3.28 ± 0.81	38.93 ± 3.23^a^	5.70 ± 0.44^a,b,c^	23.11 ± 2.78^c,d^	3.42 ± 0.46^a,b,c^
FGD-1	33.48 ± 1.55	4.06 ± 1.11	24.75 ± 1.48^b,c,d^	6.28 ± 0.25^a^	38.08 ± 3.32^a^	3.85 ± 0.02^a,b^
FGD-2	31.84 ± 0.71	3.95 ± 1.10	27.47 ± 2.95^b,c^	5.32 ± 0.14^b,c^	27.37 ± 4.31^b,c,d^	3.47 ± 0.30^a,b,c^
FGD-3	35.14 ± 2.29	4.14 ± 1.10	24.24 ± 0.60^b,c,d^	5.15 ± 0.67^c^	26.70 ± 1.39^b,c,d^	3.93 ± 0.05^a,b^

Total phenolics and flavonoids are expressed as gallic acid equivalents (GAE)/g dry mass and quercetin equivalents (QE)/g dry mass, respectively. A 50% inhibition concentration (IC_50_) value corresponds to the concentration achieving 50% scavenging activity. Pyrogallol was used to generate superoxide anions, and the results of scavenging potential are expressed in the terms of inhibition of pyrogallol oxidation. The ferric reducing capacity is expressed as mmol Fe^+2^/g dry mass. Mean values with different lower case letters (a–d) in a column are significantly different (*P* < 0.05).

We further evaluated *in vitro* antioxidant activity by 2,2-diphenyl-1-picrylhydrazyl **(**DPPH), 2′-azino-bis (3-ethylbenzothiazoline-6-sulfonic acid) (ABTS), superoxide anion scavenging and ferric reducing antioxidant power (FRAP) assay. An antioxidant that vigorously donates hydrogen is considered as a potent scavenger of DPPH^●^ with high antioxidant potential. We observed that HGD-2 was a strong scavenger of DPPH^●^ with the least 50% inhibitory concentration (IC_50_) value (20.39 ± 0.55 mg/mL) (Table 4). Further, the results of the ABTS^●+^ scavenging assay suggest that all the samples can effectively scavenge the cationic radical (ABTS^●+^). The best ABTS^●+^ scavenging activity was noted for FGD-3 with minimum IC_50_ value (5.15 ± 0.67 mg/mL). Besides, all the *Doenjang* samples showed a great promise to scavenge superoxide anions by effectively reducing the oxidation of pyrogallol. However, the best activity was displayed by GGD-10 with a least IC_50_ value (20.82 ± 0.83 mg/mL), followed by HGD-3 (23.11 ± 2.78 mg/mL). It has been well established that antioxidants can act as reductants by taking part in the redox reaction and thus have an effect on oxidants^26^. Therefore, we investigated the electron donating capacity of the *Doenjang* reflecting its reducing power using FRAP assay and observed a considerable activity of all the samples. Similar to DPPH assay, HGD-2 proved most efficient reducing agent, with 3.98 ± 0.20 mmol Fe^+2^/g ferric reducing capacity (Table 4). Results are in good agreement with the published reports showing the antioxidant potential of *Doenjang* prepared by the defined starter culture^27^.

### Anti-proliferative activity of *Doenjang* samples

**Figure 1:**
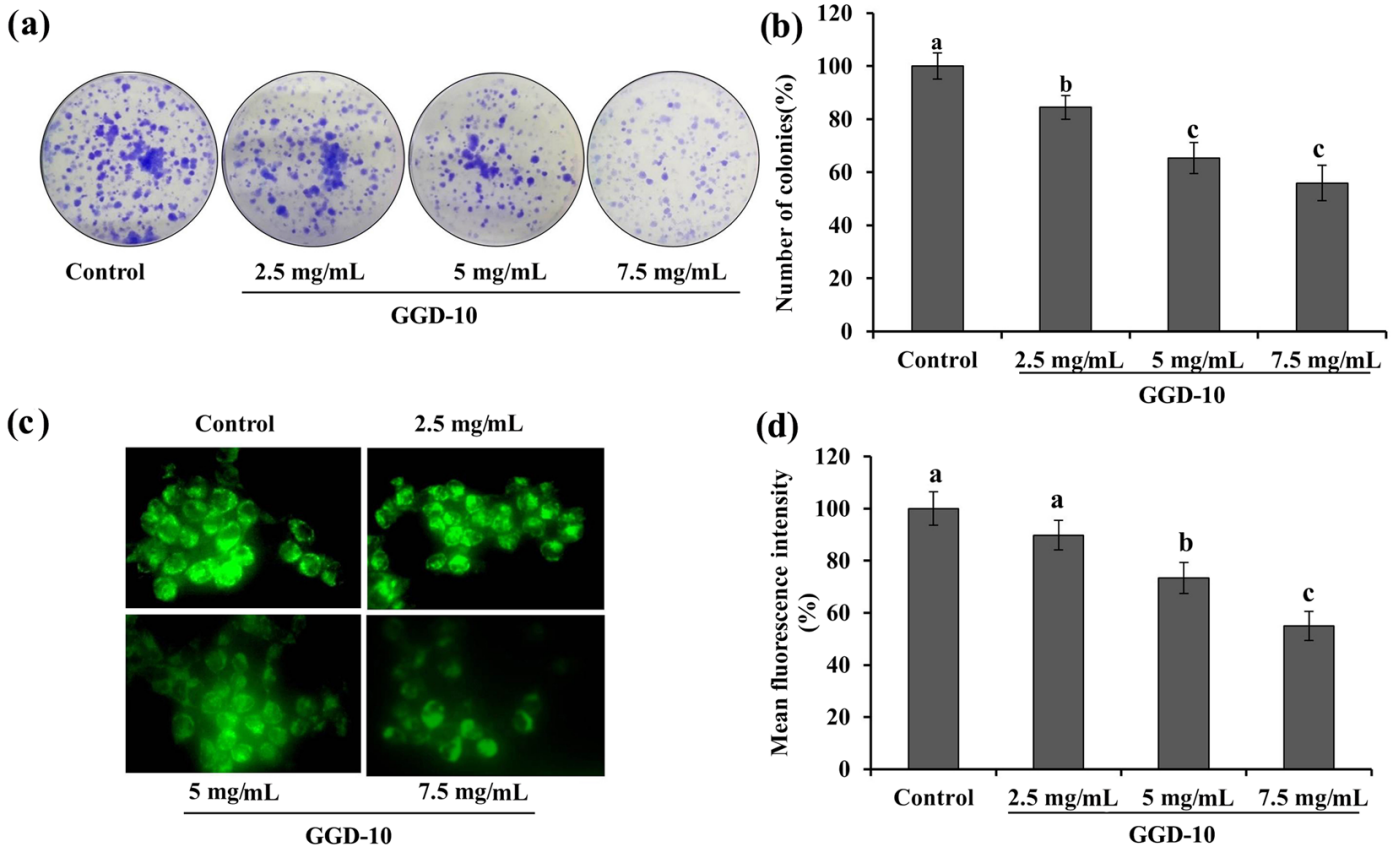
Effect of various concentrations (2.5 mg/mL, 5 mg/mL and 7.5 mg/mL) of 10% fresh garlic-supplemented *Doenjang* (GGD-10) on HT29 cells. **(a)** and **(b)** Clonogenic assay. Untreated cells acted as the control. **(c)** Mitochondrial membrane potential was evaluated using Rhodamine 123 staining. Images were captured using an epifluorescence microscope at 40× magnification. **(d)** Fluorescence intensity was determined using the Image J software. Each value in the bar graph represents the mean value of three independent experiments. Values with different lower case letters (a–c) are significantly different (*P* < 0.05).

Cancer is the most fatal disease increasing worldwide at a rapid pace. The reasons underlying the rapid progression include lifestyle, poor diet and obesity^28,29^. Therefore, the consumption of balanced hygienic food is critical as a preventive measure against cancer. A foodstuff that not only provides the essential nutrients but also has some anticancer property is much of interest as a preventive agents. Hence, we evaluated the anticancer activity of *Doenjang* against three different cancerous cell lines and observed a substantial effect (Table 5). FGD-3 showed the highest cytotoxicity against gastric adenocarcinoma (AGS) and human lung adenocarcinoma (A549) cell lines as evident by the lower IC_50_ value (7.66 ± 0.53 mg/mL and 7.82 ± 0.34 mg/mL, respectively), which was 1.5 and 1.8-fold lower as compared to the control (Table 5). Interestingly GGD-10 showed the highest cytotoxicity against colorectal adenocarcinoma (HT29) with the least IC_50_ value (10.98 ± 1.59 mg/mL). We believed that the anti-proliferative potential of the *Doenjang* is due to the presence of diverse phytochemicals in it. Importantly, phenolics and flavonoids are well known for imparting many biological activities including anticancer activity^30,31^. Results are in agreement with the published report suggesting the anticancer and antimetastatic properties of the *Doenjang*^32^. All garlic-supplemented *Doenjang* showed better activity than the control, indicating a critical role of garlic in anticancer activity, which also corroborates the results of a previous study demonstrating a potential role of garlic against various cancer cells^33^. Fresh garlic-supplemented sample (GGD-10) proved most effective against colon cancer cells (HT29) and thus selected as a representative to evaluate the clonogenic assay and the effect on mitochondrial membrane potential. Finding of the clonogenic assay revealed a significant (*P* < 0.05) dose-dependent reduction of HT29 cell proliferation and differentiation into colonies (Fig. 1a,b).

**Table 5:** Anticancer potential of garlic-supplemented *Doenjang* against three different cancerous cell lines.

*Doenjang* sample	50% inhibition concentration (mg/mL)		
	AGS	A549	HT29
Control	11.50 ± 0.80^a^	14.42 ± 2.41^a^	15.51 ± 1.21^b^
GGD-2	9.12 ± 1.49^b,c,d^	11.12 ± 0.75^a,b,c^	13.14 ± 0.96^b^
GGD-6	10.19 ± 1.88^a,b,c^	11.99 ± 3.96^a,b^	12.83 ± 1.93^b^
GGD-10	8.58 ± 1.18^c,d^	9.24 ± 0.94^b,c^	10.98 ± 1.59^b^
HGD-1	10.81 ± 1.30^a,b^	12.25 ± 4.50^a,b^	15.07 ± 4.25^b^
HGD-2	8.21 ± 1.6^c,d^	8.71 ± 1.09^b,c^	17.84 ± 3.16^b^
HGD-3	8.16 ± 1.14^c,d^	8.28 ± 0.52^b,c^	29.88 ± 11.58^a^
FGD -1	8.67 ± 0.49^b,c,d^	8.71 ± 0.35^b,c^	31.09 ± 9.78^a^
FGD-2	8.15 ± 0.40^c,d^	8.87 ± 0.93^b,c^	13.18 ± 1.11^b^
FGD-3	7.66 ± 0.53^d^	7.82 ± 0.34^c^	15.72 ± 2.69^b^

The 50% inhibition concentration value corresponds to the concentration of *Doenjang* samples causing 50% cell death of gastric adenocarcinoma (AGS), human lung adenocarcinoma (A549) and colorectal adenocarcinoma (HT29) cell lines. Mean values with different lower case letters (a–d)  in a column are significantly different (*P* < 0.05).

Mitochondria play an important role in the cancer cell survival and death. During the stress environment, mitochondrial membrane potential changes lead to opening of mitochondrial permeability transition pore and facilitate the release of cytochrome C which activates caspase cascade and ultimately induces cellular events responsible for cell death^34^. Results of mitochondrial membrane potential in response to GGD-10 are depicted in the Fig. 1c,d. GGD-10 has a significant (*P* < 0.05) effect on the mitochondrial membrane potential of HT29 evident by a dose-dependent reduced fluorescent intensity. Minimum fluorescent intensity (54.9 ± 5.6%) was observed in the cells treated with 7.5 mg/mL GGD-10 in contrast to 100% in control (without any treatment). The results collectively highlight the anti-proliferative potential of *Doenjang*, principally mediated by altering mitochondrial membrane potential and subsequently downstream events. In support to our findings, a study carried out by Jeong *et al*.^35^ revealed the role of *Doenjang* prepared by various starter culture on the up and down regulation of pro-apoptotic (Bax) and anti-apoptotic (Bcl2), respectively, in a murine model of colitis-associated colon carcinogenesis. In addition, several published reports have indicated the role of garlic in induction of apoptosis via various molecular mechanisms^33,36^. However, to the best of our knowledge, it is the first report representing the effect of garlic-supplemented *Doenjang* on alterations in mitochondrial membrane potential and ability on colony formation.

### Quantification of biogenic amines

Safety of the food products is a primary concern before its consumption, thus evaluation of toxicants and fouling agents are the utmost steps to examine the food quality^6^. Biogenic amines are formed by microbial decarboxylation of amino acids, particularly in nutritionally poor environment^37^. The presence of excessive biogenic amines in food is an indirect indicator of microbial contamination^38^. In addition, it is also the precursor of carcinogens such as *N*-nitrosamine compounds^39^. The role of biogenic amines such as tyramine, 2-phenyethylamine and putrescine to induce migration, hypersensitivity, high blood pressure and heart failure has been well cited in the literature^12^. The high amount of the biogenic amines such as tyramines and histamine in food are considered as the anti-nutritional elements^38^. Therefore, we determined the amount of biogenic amines such as agmatine, tryptamine, 2-phenylethylamine putrescine, cadaverine, histamine, tyramine, spermidine and spermine in *Doenjang* samples at the starting day and after two and four months of fermentation. The initial amount of biogenic amines in various samples was in the range of 25.36 mg/100 g–49.85 mg/100 g (Supplementary Table S1), which was decreased in most of the samples (other than HGD-2 and FGD-1) after two months of fermentation (Table 6). After two months of fermentation, the least amount of total biogenic amines was detected in FGD-3 (28.61 mg/100 g) and the highest in HGD-3 (48.22 mg/100 g) (Table 6). A negative correlation between total biogenic amines and addition of freeze-dried garlic ($y=-\,5.92x+47.3,\,{r}^{2}=0.92$) was established in *Doenjang* samples. In contrast, a positive correlation ($y=11.24x+12.16,\,{r}^{2}=0.98$) was observed in *Doenjang* prepared by mixing various proportions of heat-dried garlic. Among the biogenic amines, histamine is considered as the most important with a noteworthy role in physiological and pathological conditions^40^. In food, histamine is considered as a serious toxicant leading to histamine poisoning (scombroid) with a symptom of illness, dizziness, headache, oral burning and sweating^41^. We observed the least value of histamine in the HGD-1 (6.34 mg/100 g) (Table 6). In all garlic-supplemented *Doenjang* other than GGD-2 (20.28 mg/100 g), the histamine level was below the amount quantified in the control (18.78 mg/100 g) (Table 6). In all samples, histamine content was considerably below its toxicity level of 500 mg/kg, at which it induces severe adverse effects on human health^12^. Interestingly, we did not observe any presence of agmatine in any of the prepared *Doenjang*. When compared with two month fermentation, level of biogenic amine was slightly increased in most of the garlic-supplemented *Doenjang* after four months of fermentation (Supplementary Table S2). However, the amount is below the level of toxicity recommended for the foods^12^. Our results are consistent with the previous published report exhibiting the biogenic amine level in the different *Doenjang* samples^39^, however, unlike to the report, we noticed the low level of biogenic amines, suggesting their safety to consume.

**Table 6:** Quantification of biogenic amines in *Doenjang* samples prepared with various combinations of garlic after 2 months of fermentation (mg/100 g).

Biogenic amine	*Doenjang* samples									
	Control	GGD-2	GGD-6	GGD-10	HGD-1	HGD-2	HGD-3	FGD-1	FGD-2	FGD-3
Agmatine	0.00	0.00	0.00	0.00	0.00	0.00	0.00	0.00	0.00	0.00
Tryptamine	4.58	4.37	2.95	3.31	2.30	3.73	4.32	3.49	3.42	2.22
2-Phenylethylamine	2.37	1.81	1.49	3.36	1.73	2.94	3.40	2.18	2.34	2.02
Putrescine	6.48	5.02	6.78	10.11	4.55	6.95	9.64	6.51	3.69	2.88
Cadaverine	0.91	0.86	1.02	0.93	4.95	0.99	0.95	0.79	0.85	0.79
Histamine	18.78	20.28	13.33	15.48	6.34	13.32	17.84	12.53	16.87	11.60
Tyramine	7.21	4.98	1.61	6.66	0.97	6.17	7.96	5.58	6.16	4.99
Spermidine	1.88	1.92	1.89	1.89	1.89	1.87	1.89	1.87	1.90	1.89
Spermine	4.52	2.23	2.22	2.22	0.00	0.00	2.22	0.00	2.24	2.22
**Total**	46.73	41.47	31.30	31.30	22.74	35.96	48.22	40.45	37.36	28.61

### Determination of aflatoxin

Aflatoxins are highly toxic secondary metabolites produced by fungal species and considered as the most potent food contaminants causing severe threat to human and animals^42^. Moreover, aflatoxin plays an active role in inducing cancer^43^. As the guidelines of Korea Food and Drug Administration, 10 ppb aflatoxin in food samples is considered hazardous^44^. More than 20 types of aflatoxin have been identified, the predominant among which is aflatoxin B1, which is activated in the liver by the action of cytochrome p450 and converted into aflatoxin B1-8, 9-epoxide, a potent renal carcinogen^42^. The main sources of aflatoxin poisoning are the intake of the contaminated food, therefore, a food free from aflatoxin proves its safety towards consumers. We determined aflatoxin level in *Doenjang* samples using Neogen Veratox kit, with 5 ppb as the minimum limit of quantification. The outcome of the study revealed an absence of aflatoxin in all the *Doenjang* samples and attests the safety of *Doenjang* to consume (Table 7).

**Table 7:** Determination of total aflatoxins, aerobic and foodborne pathogenic bacterial count in *Doenjang* samples prepared with various form of garlic.

*Doenjang* sample	Aflatoxins (ppb)	Total aerobic bacteria (log CFU/g)	Pathogenic bacteria (log CFU/g)		
			*Salmonella* species	*Staphylococcus aureus*	*Bacillus cereus*
Control	—^a)^	6.22 ± 1.62	—	—	—
GGD-2	—	7.61 ± 1.82	—	—	—
GGD-6	—	7.52 ± 1.78	—	—	—
GGD-10	—	7.60 ± 1.75	—	—	—
HGD-1	—	7.57 ± 1.60	—	—	1.0 ± 0.0
HGD-2	—	7.43 ± 1.64	—	—	1.0 ± 0.0
HGD-3	—	7.59 ± 1.73	—	—	—
FGD-1	—	7.41 ± 1.77	—	—	—
FGD-2	—	7.55 ± 1.72	—	—	—
FGD-3	—	6.95 ± 1.58	—	—	1.0 ± 0.0

^a)^Not detected.

### Bacterial enumeration in *Doenjang*

Presence of microbes in the fermented food is very important to decide the fate of the product. In the present study, consortia of microbes were evolved in the soybean paste that finally fermented into the *Doenjang*. Results showed the presence of high aerobic bacterial count between 6.22 ± 1.62 log CFU/g and 7.61 ± 1.82 log CFU/g (Table 7) in the fermented product. Interestingly, higher aerobic bacterial count was noticed in garlic-supplemented *Doenjang* than in the control. In all the prepared *Doenjang*, the highest aerobic bacterial count was observed in the GGD-2 (7.61 ± 1.82 log CFU/g), which was approximately 1.2-times higher than that in the control (6.22 ± 1.62 log CFU/g) (Table 7). The high number of aerobic bacteria in *Doenjang* is an index of good fermentation, which leads to conversion of complex biomolecules into simple and vital phytochemicals responsible for imparting better metabolic profile to *Doenjang*. Results are in agreement with the previous observations regarding the involvement of various microbes in *Doenjang* formation^6,11^.

There is always being a high chance of pathogenic bacteria contamination in the complexed food material when stored for a long time. Common foodborne pathogens (*Escherichia coli, Bacillus cereus, Salmonella* species and *Staphylococcus aureus*) account for huge economic and health loss every year. The severity of *Salmonella* infection can be assumed by the fact that millions of cases of gastroenteritis are reported worldwide every year, primarily by the consumption of the contaminated food^45^. To access the pathogen-free preparation of *Doenjang*, we examined the *Salmonella* species and *S. aureus* in it and found the absence of these important pathogens. There are many possible reasons for the absence of these bacteria in *Doenjang*. One common reason is the acidic pH that acts as a barrier to the survival of these pathogens^6^. However, pathogens such as *B. cereus* can grow well in acidic environment and is considered one of the most serious pathogens in fermented food, where the level larger than 5.0 log CFU/g in food potentially causes food poisoning^46^. In our study, we observed 1.0 ± 0.0 log CFU/g in HGD-1, HGD-2 and FGD-3, which are well below the toxic level (5.0 log CFU/g). The remaining samples, including the control, were free from the *B. cereus* contamination (Table 7). The finding suggests a pathogen-free preparation of *Doenjang* that could be used as a safe food.

## Conclusion

Based on the findings, we conclude that garlic has a positive impact on *Doenjang* by enhancing its nutritional and functional properties. Garlic-supplemented *Doenjang* demonstrated an enhanced level of phenolics, flavonoid and antioxidant along with the substantial anticancer activity. A high level of total free amino acids in garlic-supplemented *Doenjang* makes it nutritionally more valuable and can be used as a source of functional food. Moreover, a lower level of antinutritional biogenic amines and absence of aflatoxin suggests that it is safe to consume. Although there are many reports of *Doenjang* preparation using different starter culture and supplementation of phytochemicals, to the best of our knowledge, it is the first comprehensive study suggesting *Doenjang* preparation augmented with garlic.

## Materials and Methods

### Chemicals and reagents

All chemicals, unless otherwise stated, were of the highest quality and were used as supplied. All the standard biogenic amines (putrescine dihydrochloride, agmatine sulfate, histamine dihydrochloride, 2-phenylethylamine, tyramine hydrochloride, spermine tetrahydrochloride, tryptamine hydrochloride, spermidine trihydrochloride and cadaverine dihydrochloride) were purchased from Sigma-Aldrich (St. Louis, MO, USA). High-performance liquid chromatography (HPLC) grade ammonium acetate and acetonitrile were purchased from Merck (Damstadt, Germany).

### *Doenjang* preparation

Soybean seeds were soaked for 12 h in water, followed by boiling for 1.5 h. The cooked seeds were ground to make an even soybean paste that were eventually transferred into stainless steel square plates (25 cm length × 20 cm width × 10 cm height) to make bricks for the formation of *Meju*. The soybean bricks were incubated at ambient temperature (~28 °C) for 14 days to allow growth of natural microflora (*Meju*). The soybean bricks (*Meju*) were divided into four different groups, (i) soybean bricks mixed with 2%, 6% and 10% (w/w) paste of fresh garlic, (ii) and (iii) soybean bricks mixed with 1%, 2% and 3% (w/w) heat-dried and freeze-dried garlic, respectively, and (iv) soybean bricks without any garlic mix (control) (Table 1). Finally, 18% salt solution was added to each group and incubated at room temperature (~28 °C) for two months. After that liquid portion was separated, the solid part was further incubated for four months at ambient temperature (~28 °C) (Supplementary Fig. S1). The experiment was performed in triplicate for each group.

### pH measurement

The pH of the *Doenjang* samples was determined using a pre-calibrated pH meter per the method described by Shukla *et al*.^39^. In brief, 10 g *Doenjang* samples were homogenized in 100 mL distilled water, followed by filtration with Whatman paper No. 2 (Advantec; Tokyo, Japan). Finally, the pH of the filtered samples was measured using a pH meter (Thermo Electron Corporation; Beverly, MA, USA).

### Moisture, salt and ash content

The moisture content in the *Doenjang* samples was determined by a gravimetric method by placing 2 g sample into the preheated oven at 105 ± 2 °C until constant weight was achieved according to standard method of Association of Official Analytical Chemists^47^.

Salt content in the *Doenjang* samples was determined according to the method of the Association of Official Analytical Chemists^47^, and the results were expressed as percentage salinity.

Percentage ash content in the *Doenjang* samples was evaluated using the thermo-gravimetric method by placing 2 g sample in a crucible and heating up to 600 °C for 6 h in the muffle furnace according to standard method of the Association of Official Analytical Chemists^47^.

### Color analysis

Color of *Doenjang* samples was examined using a colorimeter (CR-300, Minolta; Osaka, Japan). The colorimeter was calibrated using a standard white plate with Hunter color values (L* = 96.43, a* = +0.03 and b* = +1.79). L* represents the lightness of the color and spans from 0 (dark) to 100 (light), a* represents the extent of red and green color in the range −60 (green) to 60 (dark red) and b* represents the intensity of yellow-blue color in the range −60 (blue) to 60 (yellow).

### Determination of free amino acids

#### Extraction of amino acids from Doenjang

Amino acids from *Doenjang* samples were extracted as described previously^6^. In brief, 100 g sample was homogenized in 150 mL ethanol (86.5%) and refluxed for 1 h at 65 °C. The suspension was filtered using Whatman paper No. 2, and the residue was re-extracted twice as mentioned previously, followed by filtration. All the filtrates were pooled and centrifuged at 4,000 × *g* for 20 min at 4 °C. The supernatant was collected and vacuum-dried using a rotatory evaporator (Eyela; Tokyo, Japan). The dried samples were dissolved in 300 mL methanol and filtered for de-saltation. The filtrate was vacuum-dried and finally dissolved in 100 mL distilled water.

#### Isolation and identification of amino acids

Free amino acids in the solution were separated by ion exchange chromatography using amberlite IR-120 (cation exchanger) and IRA-400 (anion exchanger) column. Bound amino acids were recovered by washing the column with 2 N NH_4_OH at slow flow rate. Eluted amino acids were dried and then dissolved in 2 mL 0.2 M lithium citrate buffer (Biochrom; Cambridge, England). Finally, 10 µL sample was injected into the amino acid autoanalyzer (L-8900, Hitachi; Tokyo, Japan), and the chromatogram was analyzed to quantify the presence of different amino acids.

### Estimation of total phenolics and flavonoids

For estimation of phenolics and flavonoids, 10 g *Doenjang* samples were extracted with 100 mL water by refluxing it at 70 °C for 3 h, followed by filtration with Whatman filter paper No. 2. Residue was re-extracted twice under same experimental conditions and filtered. All the filtrates were pooled and finally freeze-dried to obtain the dry powder which was used further for estimating phenolics, flavonoids, antioxidant and cytotoxic potential. Total phenolics in the aqueous extracted *Doenjang* samples were determined by Folin-Ciocalteu method^48^, while the flavonoids were estimated by the previously describe method^49^ and expressed as GAE/g dry mass and QE/g dry mass, respectively.

### Determination of *in vitro* antioxidant activity

*In vitro* antioxidant potential of *Doenjang* samples was examined by DPPH^50^, ABTS^51^, superoxide anion scavenging^52^ and FRAP assay^53^. IC_50_ was calculated by using multiple regression equation generated from increasing concentration of samples against percent scavenging. Percent scavenging at different concentrations was calculated using following equation: scavenging (%) (1 − *As*/*Ac*) × 100, where *As* is the absorbance in presence of *Doenjang* sample, while *Ac* is the absorbance of the control devoid of any sample.

### Anti-proliferative potential of *Doenjang*

#### Cell lines and cell culture

Gastric adenocarcinoma (AGS), human lung adenocarcinoma (A549) and colorectal adenocarcinoma (HT29) cell lines were purchased from American Type Culture Collection (Manassas, VA, USA). AGS and A549 cells were maintained on RPMI-1640 medium (Invitrogen; Carlsbad, CA, USA), while HT29 were cultured on DMEM media (Invitrogen) supplemented with 10% (v/v) fetal calf serum (Gibco; Grand Island, NY, USA) and 1% penicillin-streptomycin mix (Gibco) at 37 °C in 5% CO_2_ incubator.

#### 3-(4,5-Dimethylthiazol-2-yl)-2,5-diphenyltetrazolium bromide (MTT) assay

Anti-proliferative potential of *Doenjang* samples was evaluated by MTT assay against three different human cancer cell lines of AGS, A549 and HT29. All the three different cancer cell lines were seeded individually in separate 96-well   cell culture plates at the density of 1 × 10^4^ cells/well and incubated for 24 h. After incubation, cells were treated individually with each aqueous extracted *Doenjang* samples at different concentrations (0.0 mg/mL, 0.01 mg/mL, 0.1 mg/mL, 1.0 mg/mL and 10.0 mg/mL) and re-incubated for 24 h. After incubation, cells were treated with MTT solution (0.5 mg/mL) to produce dark blue color formazan, which was subsequently dissolved in the dimethyl sulfoxide. Finally, absorbance was measured at 540 nm in a microplate reader (Tecan; Mannedorf, Switzerland), and results were expressed as percent viable cells.

#### Clonogenic assay

For clonogenic assay, 8 × 10^2^ HT29 cells/well were seeded in 12 well-culture plate, followed by the treatment of various concentrations (0.0 mg/mL, 2.5 mg/mL, 5 mg/mL and 7.5 mg/mL) of 3% freeze-dried garlic added *Doenjang*. Cells were allowed to grow for 7 days in a CO_2_ incubator at 37 °C. Finally, media were aspirated and cells (clones) were washed with 0.1 M phosphate buffer saline (PBS) (pH 7.4) and stained with 0.5% crystal violet.

#### Mitochondrial membrane potential

Effect of *Doenjang* samples on the mitochondrial membrane potential of HT29 was evaluated by a cell permeable green fluorescent Rhodamine 123 dye^34^. In brief, 1 × 10^4^ cells/well were seeded into 24-well cell culture plate, followed by the treatment of various concentrations (0.0 mg/mL, 2.5 mg/mL, 5 mg/mL and 7.5 mg/mL) of 10% fresh ground garlic-supplemented *Doenjang*. Finally, cells were washed with 0.1 M PBS and stained for 30 min with Rhodamine 123 (1 µg/mL). After washing twice with 0.1 M PBS, fluorescent images were captured under epi-fluorescence microscope (Nikon; Kanagawa, Japan), and fluorescent intensity was quantified by Image-J software (National Institutes of Health; Bethesda, Maryland, USA).

### Determination of biogenic amines

#### Extraction of biogenic amines

Biogenic amines were extracted from *Doenjang* samples by the method adopted by Shukla *et al*.^39^. In brief, 5 g *Doenjang* samples were homogenized with 10 mL of 0.4 M perchloric acid, followed by centrifugation at 4,000 × *g* for 10 min at 4 °C. The supernatant was recovered, and the pellet was re-extracted twice with 0.4 M perchloric acid, followed by centrifugation. All the collected supernatants were polled, filtered through Whatman paper No. 1 and used for HPLC analysis after derivatization with dansyl chloride.

#### Derivatization of extracted biogenic amines

Derivatization of extracted biogenic amines was carried out by adopting the previously described method^39^. In brief, 1 mL of each extracted biogenic amine suspension was mixed with 200 µL of 2 M NaOH, 300 µL of NaHCO_3_ (saturated solution) and 2 mL of 0.04 M dansyl chloride, followed by 45 min incubation at 40 °C. After the incubation, 100 µL of 25% NH_4_OH was added to stop the reaction, and removal of excess dansyl chloride was succeeded by the addition of acetonitrile to make the volume up to 5 mL. Finally, the mixture was centrifuged at 2,500 × *g* for 5 min and filtered through 0.2 µm syringe filter (Woongki Science Co.; Seoul, Korea). The filtered samples were stored at −25 °C until use for HPLC.

#### Separation and quantification of biogenic amines by HPLC

Separation and quantification of derivatized biogenic amines were carried out using HPLC analysis (Hewlett Packard series-1100, Agilent Technologies; Santa Clara, CA, USA) equipped with a UV-visible spectrophotometer as a detector. Separation was carried out by injecting 10 µL sample into a C_18_ column (5 µm pore size, 4.6 mm internal diameter, 250 mm length) using 0.1 M ammonium acetate and acetonitrile as mobile phase with gradient programming up to 35 min. Separation was commenced at 30 °C with a constant mobile phase flow rate of 1 mL/min. Separated biogenic amines were analyzed at 254 nm. Biogenic amines in the samples were identified and quantified by comparing with the known standards.

### Determination of aflatoxin

Aflatoxin was extracted and quantified from *Doenjang* samples as per the instructions of the manufacturer (Neogen Corp.; Lansing, MI, USA), supplied with ELISA-based aflatoxin detection kit (Veratox for total aflatoxin). In brief, 10 g sample was homogenized with 50 mL of 70% methanol, followed by filtration with Whatman filter paper No. 2. The filtrate was directly used to detect aflatoxin using ELISA kit. After sufficient antigen (aflatoxin)-antibody interaction, chromogenic substrate was added, and then an absorbance of the colored product was measured at 650 nm using microplate reader (Infinite M200, Tecan; Mannedorf, Switzerland). Quantification of aflatoxin was made by Neogen’s Veratox data reduction software by converting absorbance into concentration against reference standards.

### Enumeration of aerobic bacteria in *Doenjang* samples

Presence of total aerobic bacteria was examined by homogenizing 10 g *Doenjang* sample in 90 mL sterilized saline (0.85% NaCl). The suspension was serially diluted up to 10^−7^, and then the100 µL of each dilution was spread over the plate count agar medium (Oxoid; Hampshire, England) and incubated at 30 °C for 24 h. In addition, the presence of common food pathogens (*B. cereus, S. aureus* and *Salmonella* species) was also determined.

### Detection of *Bacillus cereus*

Ten gram sample was homogenized into 90 mL sterilized saline (0.85% NaCl) and serially diluted up to 10^−5^. One hundred microliter of each dilution was spread over mannitol-egg yolk-polymyxin agar plate (MYP) (Oxoid; Hampshire, England) and incubated at 30 °C for 24 h. Presence of *B. cereus* was evaluated by the appearance of characteristic pink colonies with transparent margins. Confirmatory test was carried out by streaking the positive isolates from MYP medium into nutrient agar plate, followed by identification using API 50 CHB kit (bioMerieux; Marcy I’ Etoile, France). To exclude any rare possibility of the isolate as *B. thuringiensis,* a microscopic examination for crystal proteins have been done as per the method suggested by USFDA^54^.

### Detection of *Salmonella* species

For detection of *Salmonella* species, 10 g sample was homogenized in 90 mL peptone water and incubated at 35 °C for 18 h. Further, 100 µL suspension was transferred into 10 mL of Rappaport Vassiliadis broth (Merck; Darmstadt, Germany) and incubated at 42 °C for 24 h. At last, 100 µL suspension was spread over MacConkey agar (Becton, Dickinson and Company; Sparks, MD, USA) plate and incubated at 35 °C for 24 h. Presence of *Salmonella* species were detected by the appearance of characteristic colorless colonies.

### Detection of *Staphylococcusaureus*

Ten gram sample was homogenized in 90 mL tryptic soy broth (Becton, Dickinson and Company) with 10% NaCl and incubated at 35 °C for 16 h. Finally, 100 µL suspension was spreaded over Baird Parker agar plate (Oxoid; Hampshire, England) supplemented with egg yolk-tellurite emulsion (Oxoid). The plates were incubated at 35 °C for 24 h. Characteristic black color colonies were examined for the presence of *S. aureus*.

### Statistical analysis

All experiments were carried out in triplicates. The results were expressed as mean ± standard deviation of three independent experiments. Multiple comparisons were performed using one-way analysis of variance, followed by Duncan test for post hoc analysis using SPSS16 software (SPSS Inc.; Chicago, IL, USA). *P* < 0.05 were considered statistically significant.

## Supplementary information

Dataset 1

## Data Availability

The authors declare that all the data supporting the finding of this study are available within the article and from the corresponding author on reasonable request.
