# Supplementary material for: Garlic augments the functional and nutritional behavior of Doenjang, a traditional Korean fermented soybean paste
Source: Sci Rep. 2019 Apr 1;9:5436. doi: 10.1038/s41598-019-41691-3 (PMC6443795; doi:10.1038/s41598-019-41691-3)
Supplement: Supplementary file 1 — Dataset 1 [file 41598_2019_41691_MOESM1_ESM.docx]

**Supplementary data set 1**

**Garlic augments the functional and nutritional behavior of *Doenjang*, a traditional Korean fermented soybean paste**

**Ashutosh Bahuguna^a,¶^, Shruti Shukla^b,¶^, Jong Suk Lee^c^, Vivek K. Bajpai^b^,** So-Young Kim^d^, **Yun Suk Huh^e,*^, Young-Kyu Han^b,**^, Myunghee Kim^a,^*****

^a^Department of Food Science and Technology, Yeungnam University, Gyeongsan, Gyeongsangbuk-do 38541, Republic of Korea

^b^Department of Energy and Materials Engineering, Dongguk University-Seoul, 30 Pildong-ro 1-gil, Seoul 04620, Republic of Korea

^c^Division of Food & Nutrition and Cook, Taegu Science University, Daegu 41453, Republic of Korea

^d^Department of Agrofood Resources, National Institute of Agricultural Sciences, Rural Development Administration, 166 Nongsaengmyeong-ro, Iseo-myeon, Wanju, Jellabuk-do 55365, Republic of Korea

^e^Department of Biological Engineering, Biohybrid Systems Research Center (BSRC), Inha University, 100 Inha-ro, Nam-gu, Incheon 22212, Republic of Korea

**Running head:** Quality analysis of garlic-supplemented *Doenjang*

**^¶^These authors contributed equally to this work.**

***Co-corresponding author**

yunsuk.huh@inha.ac.kr (Y.S. Huh)

****Co-corresponding author**

ykenergy@dongguk.edu (Y.-K. Han)

*****Corresponding author:** [foodtech@ynu.ac.kr](mailto:foodtech@ynu.ac.kr) (M. Kim)


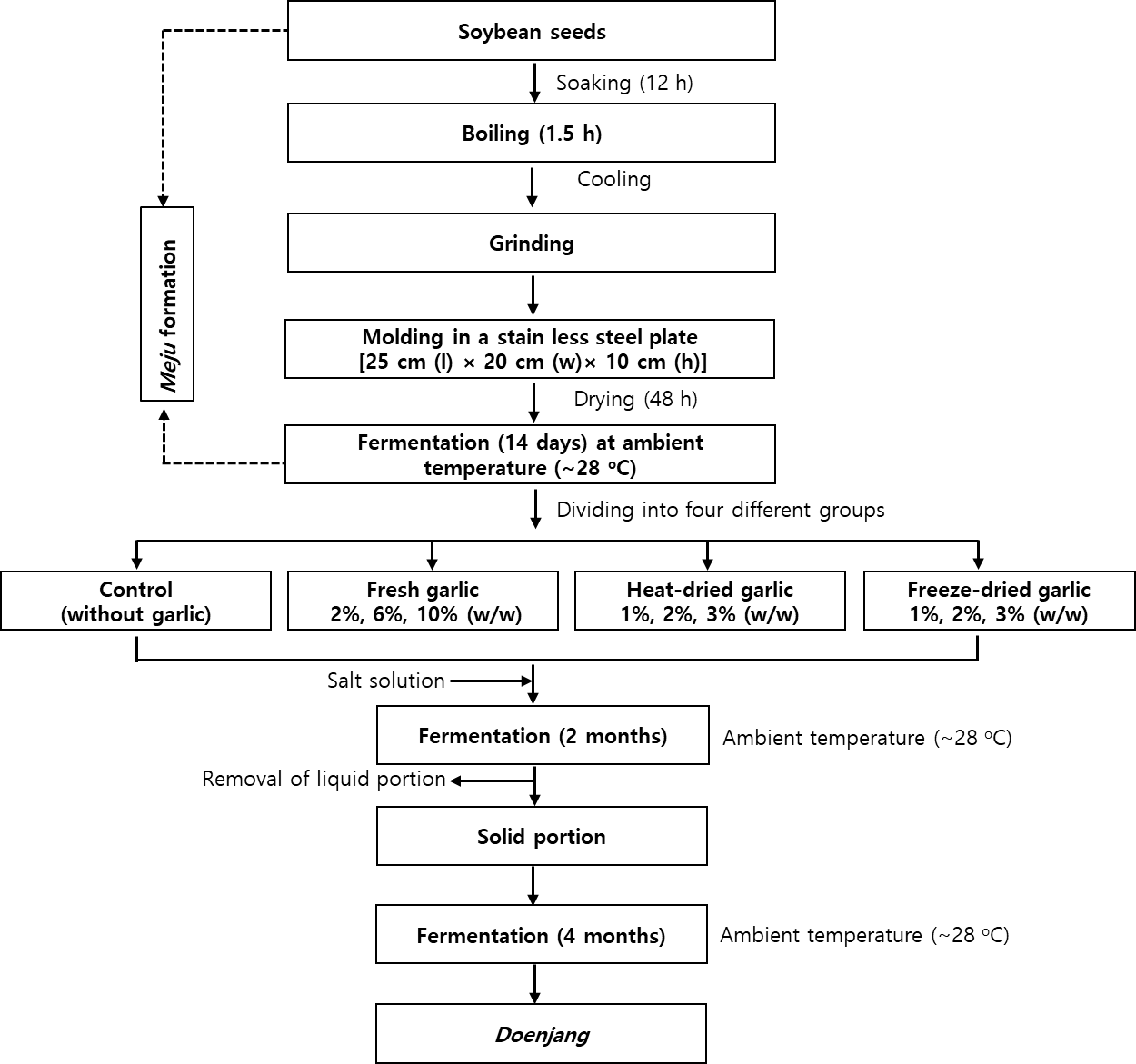


**Figure S1.** Systemic method for the preparation of *Doenjang.*


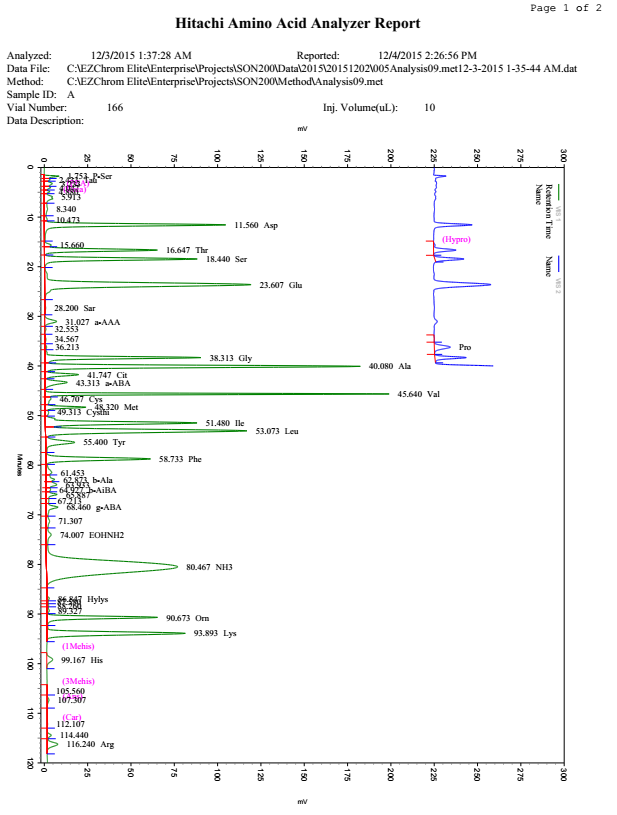


**Figure S2.** Amino acid analyzer chromatogram of *Doenjang* control (without any garlic).


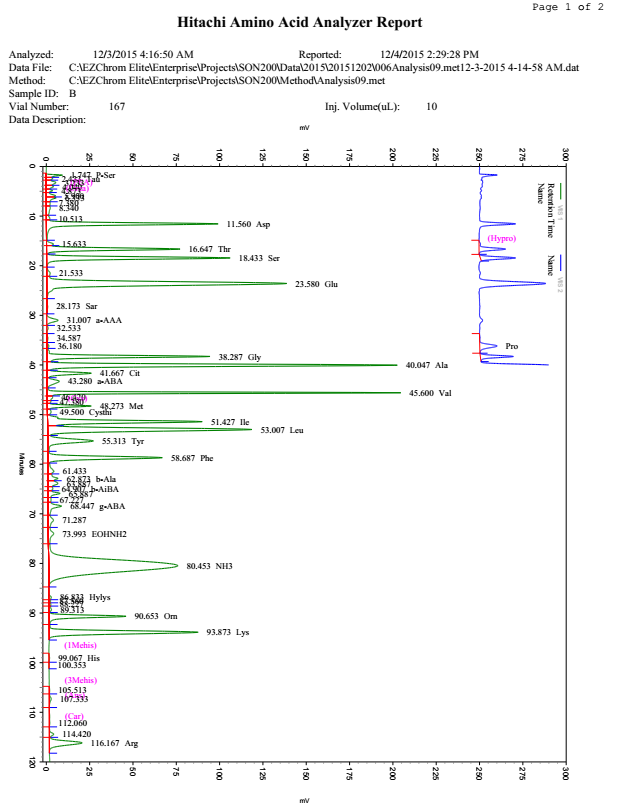


**Figure S3.** Amino acid analyzer chromatogram of 2% fresh garlic*-*supplemented *Doenjang* (GGD-2).


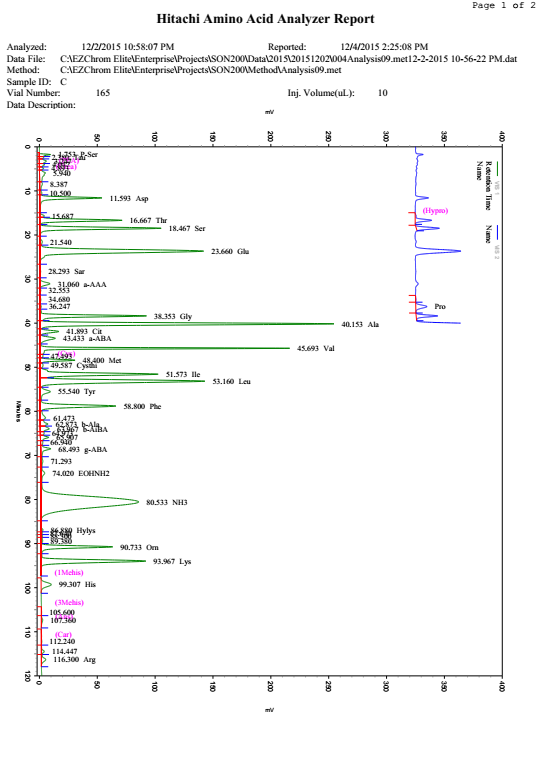


**Figure S4.** Amino acid analyzer chromatogram of 6% fresh garlic-supplemented *Doenjang* (GGD-6).
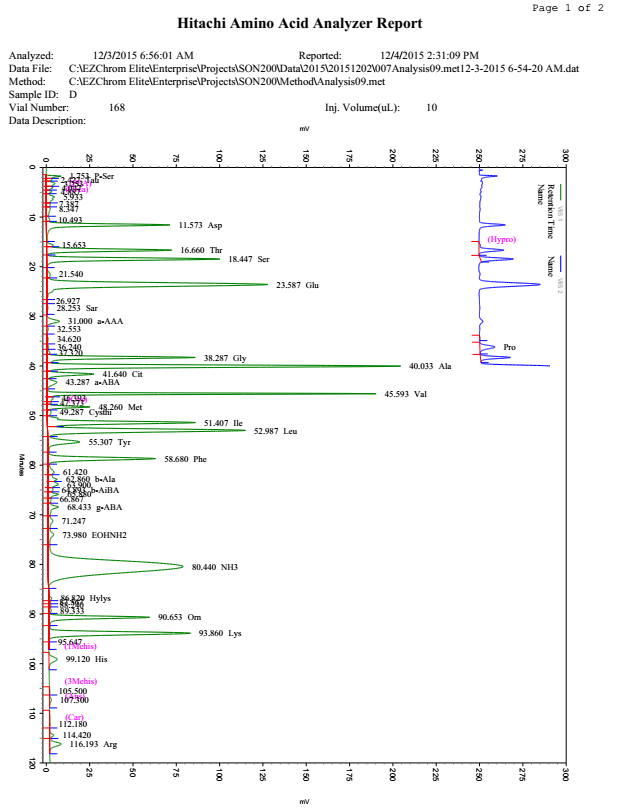


**Figure S5.** Amino acid analyzer chromatogram of 10% fresh garlic*-*supplemented *Doenjang* (GGD-10).
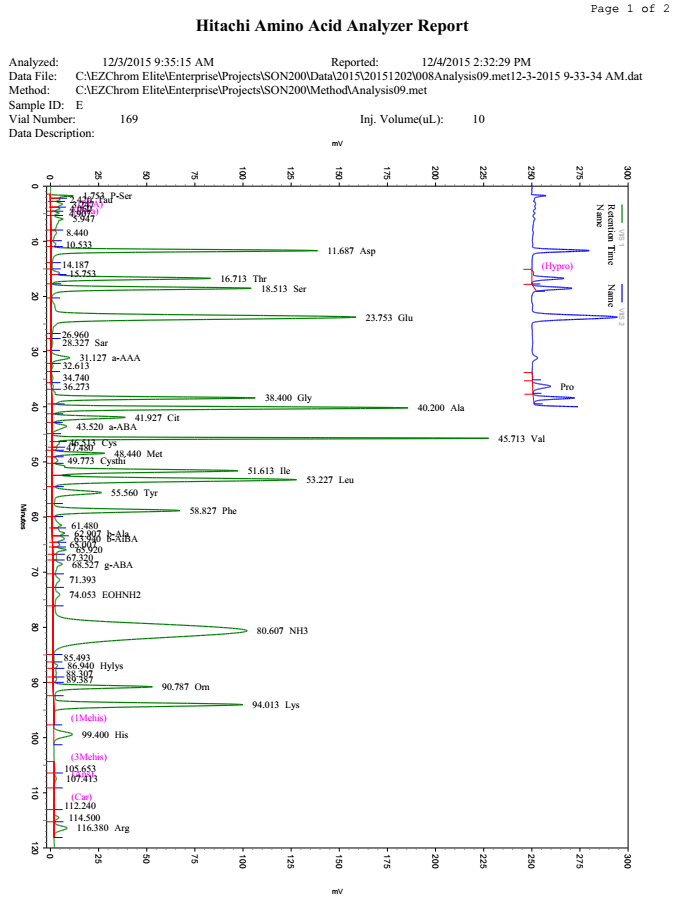


**Figure S6.** Amino acid analyzer chromatogram of 1% heat-dried garlic-supplemented *Doenjang* (HGD-1).


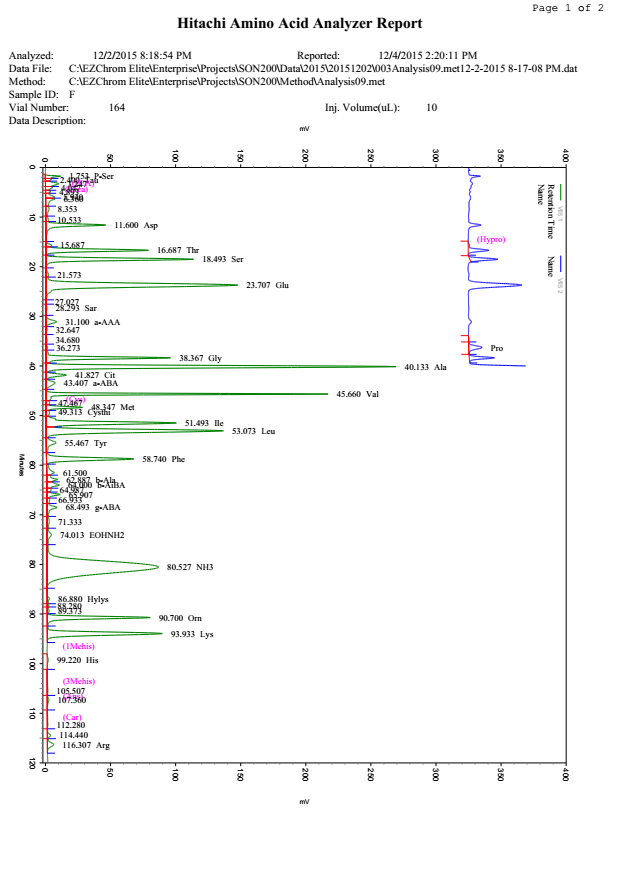


**Figure S7.** Amino acid analyzer chromatogram of 2% heat-dried garlic-supplemented *Doenjang* (HGD-2).


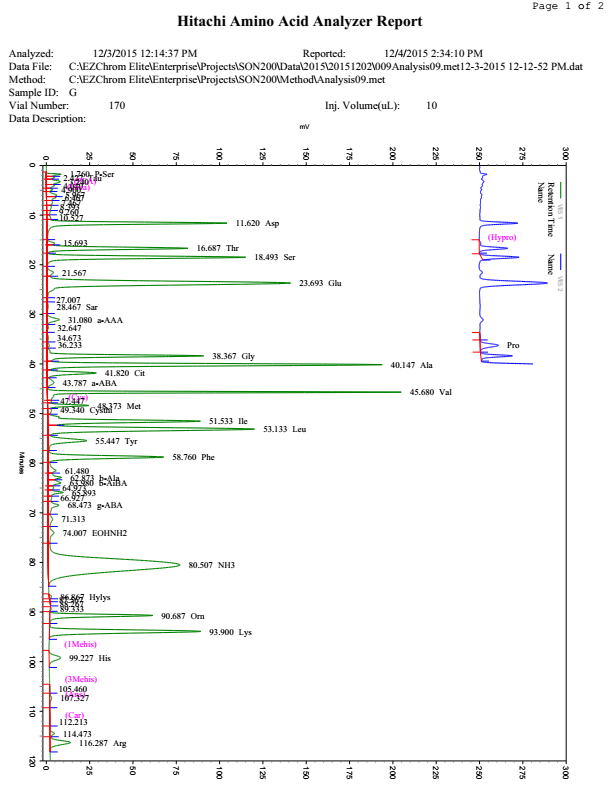


**Figure S8.** Amino acid analyzer chromatogram of 3% heat-dried garlic-supplemented *Doenjang* (HGD-3).


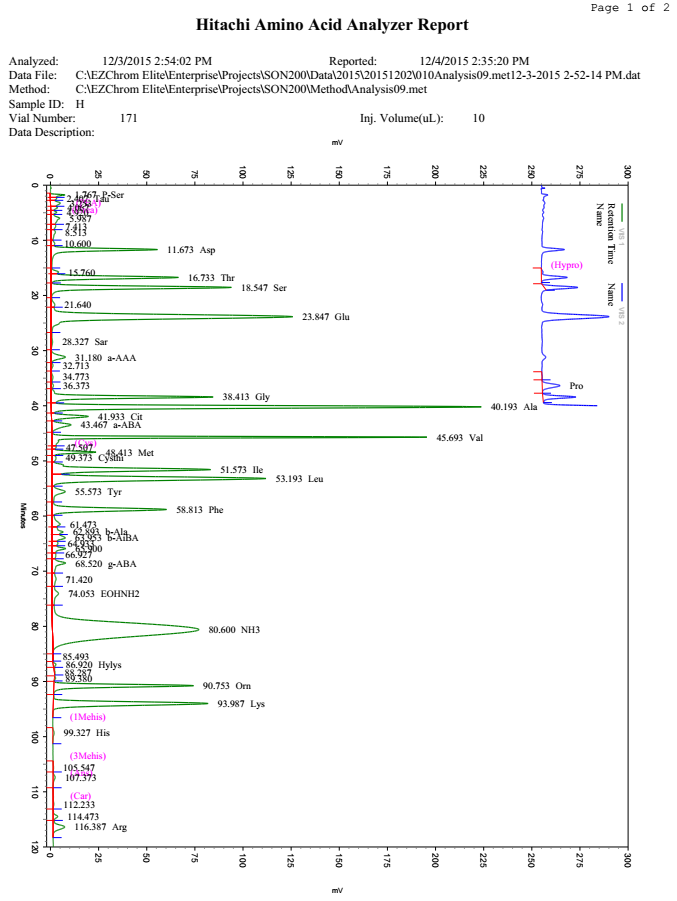


**Figure S9.** Amino acid analyzer chromatogram of 1% freeze-dried garlic-supplemented *Doenjang* (FGD-1).


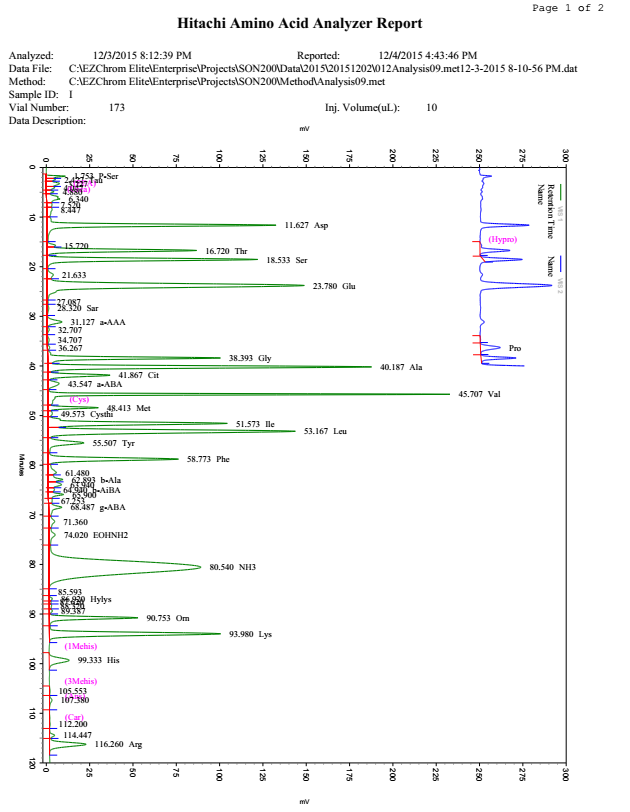


**Figure S10.** Amino acid analyzer chromatogram of 2% freeze-dried garlic-supplemented *Doenjang* (FGD-2).


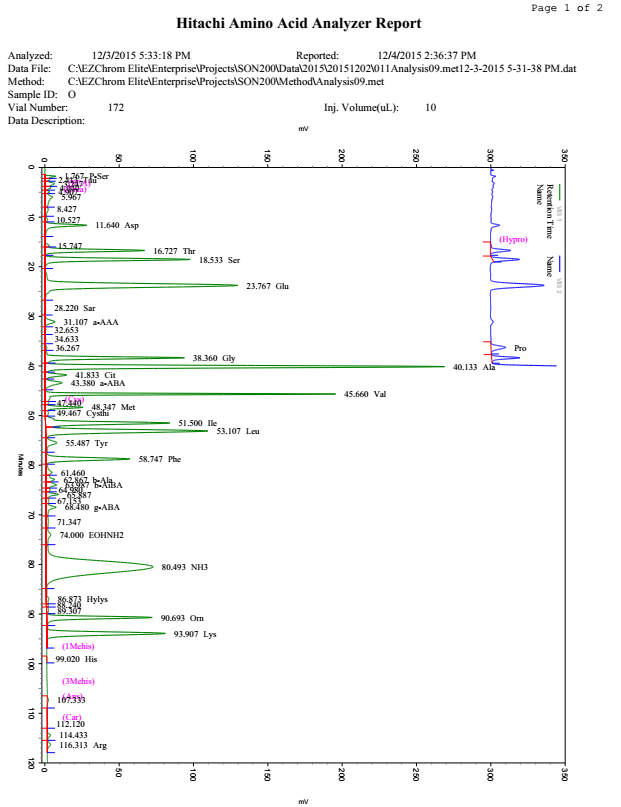


**Figure S11.** Amino acid analyzer chromatogram of 3% freeze-dried garlic-supplemented *Doenjang* (FGD-3).


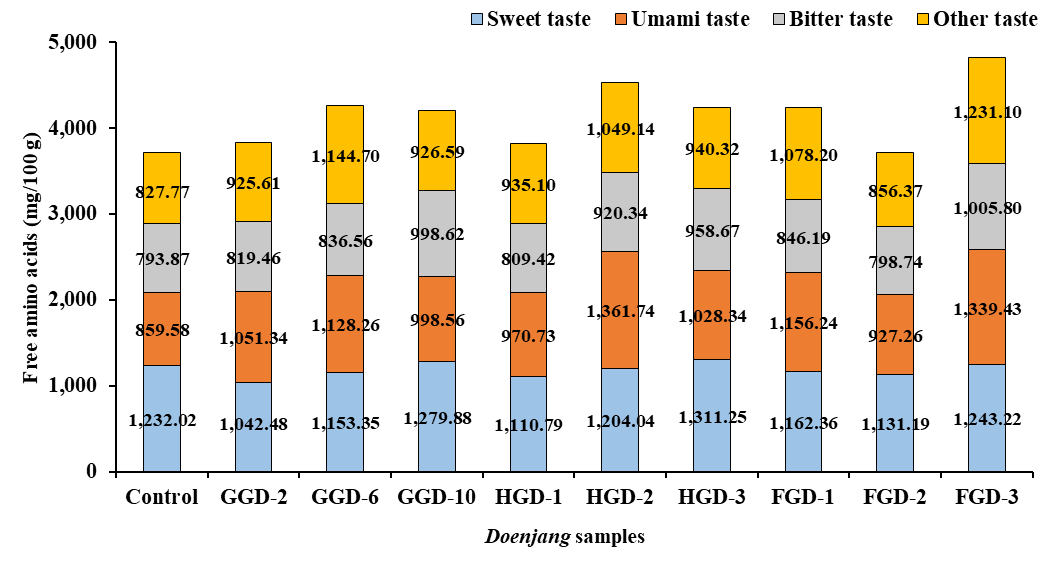


**Figure S12.** A composition of free amino acids based on their taste in different *Doenjang* samples. Sweet taste represents the sum of lysine, glycine, alanine, serine and threonine. Umami taste represents the sum of glutamic acid, aspartic acid and cysteine, while bitter taste represents the sum of isoleucine, leucine and methionine. The other taste is the sum of phenylalanine, valine, arginine, proline, tyrosine and histidine.

**Table S1.** Quantification of biogenic amines in *Doenjang* samples prepared with various combinations of garlic (mg/100 g).

| **Biogenic amine** | ***Doenjang* samples** | | | | | | | | |  |
| --- | --- | --- | --- | --- | --- | --- | --- | --- | --- | --- |
|  | **Control** | **GGD-2** | **GGD-6** | **GGD-10** | **HGD-1** | **HGD-2** | **HGD-3** | **FGD-1** | **FGD-2** | **FGD-3** |
| Agmatine | 0.00 | 0.00 | 0.00 | 0.00 | 0.00 | 0.00 | 0.00 | 0.00 | 0.00 | 0.00 |
| Tryptamine | 5.53 | 4.43 | 3.26 | 2.08 | 2.41 | 2.65 | 4.57 | 4.08 | 3.95 | 2.92 |
| 2-Phenylethylamine | 3.53 | 2.34 | 1.60 | 2.29 | 1.78 | 2.33 | 3.66 | 2.47 | 2.40 | 2.55 |
| Putrescine | 14.46 | 9.06 | 14.05 | 7.36 | 6.77 | 3.55 | 11.76 | 4.31 | 4.37 | 5.34 |
| Cadaverine | 1.13 | 1.02 | 1.14 | 0.94 | 1.09 | 0.92 | 1.05 | 0.93 | 0.95 | 0.94 |
| Histamine | 18.04 | 16.71 | 13.10 | 13.17 | 6.86 | 12.34 | 19.40 | 15.10 | 17.45 | 8.13 |
| Tyramine | 7.17 | 7.76 | 3.57 | 9.02 | 4.23 | 6.05 | 7.65 | 6.40 | 6.60 | 6.57 |
| Spermidine | 0.00 | 0.00 | 0.01 | 0.00 | 0.00 | 0.00 | 0.00 | 0.00 | 0.00 | 0.00 |
| Spermine | 0.00 | 2.23 | 2.23 | 1.12 | 2.22 | 1.11 | 1.11 | 2.23 | 2.24 | 2.23 |
| **Total** | 49.85 | 43.55 | 38.97 | 38.97 | 25.36 | 28.96 | 49.20 | 35.52 | 37.96 | 28.67 |

**Table S2.** Quantification of biogenic amines in *Doenjang* samples prepared with various combinations of garlic after 4 months of fermentation (mg/100 g).

| **Biogenic amine** | ***Doenjang* samples** | | | | | | | | |  |
| --- | --- | --- | --- | --- | --- | --- | --- | --- | --- | --- |
|  | **Control** | **GGD-2** | **GGD-6** | **GGD-10** | **HGD-1** | **HGD-2** | **HGD-3** | **FGD-1** | **FGD-2** | **FGD-3** |
| Agmatine | 0.00 | 0.00 | 0.00 | 0.00 | 0.00 | 0.00 | 0.00 | 0.00 | 0.00 | 0.00 |
| Tryptamine | 5.29 | 4.20 | 3.17 | 3.17 | 2.09 | 3.90 | 4.73 | 3.99 | 3.39 | 2.46 |
| 2-Phenylethylamine | 2.41 | 2.12 | 2.90 | 2.90 | 1.01 | 2.89 | 3.19 | 2.30 | 2.20 | 2.01 |
| Putrescine | 8.76 | 13.18 | 12.35 | 12.35 | 8.52 | 7.90 | 10.43 | 3.58 | 4.77 | 3.30 |
| Cadaverine | 1.10 | 1.13 | 1.13 | 1.13 | 1.38 | 1.24 | 1.22 | 1.11 | 1.10 | 1.17 |
| Histamine | 18.98 | 17.80 | 13.82 | 13.82 | 8.10 | 12.56 | 18.81 | 14.58 | 16.63 | 12.77 |
| Tyramine | 7.01 | 6.71 | 8.75 | 8.75 | 4.72 | 2.95 | 7.35 | 5.73 | 6.71 | 5.44 |
| Spermidine | 2.05 | 2.17 | 1.01 | 1.01 | 0.00 | 6.97 | 2.07 | 0.00 | 0.92 | 1.84 |
| Spermine | 0.00 | 0.00 | 0.00 | 0.00 | 0.00 | 0.00 | 0.00 | 0.00 | 2.71 | 5.36 |
| **Total** | 45.60 | 47.31 | 43.13 | 34.62 | 25.82 | 38.42 | 47.80 | 31.28 | 38.43 | 34.35 |
